# Short-Term Dietary Intervention with Whole Oats Protects from Antibiotic-Induced Dysbiosis

**DOI:** 10.1128/spectrum.02376-23

**Published:** 2023-07-13

**Authors:** Stephen K. Costa, Katherine Antosca, Chapman N. Beekman, Rachel L. Peterson, Swathi Penumutchu, Peter Belenky

**Affiliations:** a Department of Molecular Microbiology and Immunology, Brown University, Providence, Rhode Island, USA; University of Minnesota, Twin Cities

**Keywords:** antibiotic-induced dysbiosis, dysbiosis, gut microbiome, microbiome

## Abstract

Antibiotic-induced gut microbiome dysbiosis (AID) is known to be influenced by host dietary composition. However, how and when diet modulates gut dysbiosis remains poorly characterized. Thus, here, we utilize a multi-omics approach to characterize how a diet supplemented with oats, a rich source of microbiota-accessible carbohydrates, or dextrose impacts amoxicillin-induced changes to gut microbiome structure and transcriptional activity. We demonstrate that oat administration during amoxicillin challenge provides greater protection from AID than the always oats or recovery oats diet groups. In particular, the group in which oats were provided at the time of antibiotic exposure induced the greatest protection against AID while the other oat diets saw greater effects after amoxicillin challenge. The oat diets likewise reduced amoxicillin-driven elimination of *Firmicutes* compared to the dextrose diet. Functionally, gut communities fed dextrose were carbohydrate starved and favored respiratory metabolism and consequent metabolic stress management while oat-fed communities shifted their transcriptomic profile and emphasized antibiotic stress management. The metabolic trends were exemplified when assessing transcriptional activity of the following two common gut commensal bacteria: Akkermansia muciniphila and Bacteroides thetaiotaomicron. These findings demonstrate that while host diet is important in shaping how antibiotics effect the gut microbiome composition and function, diet timing may play an even greater role in dietary intervention-based therapeutics.

**IMPORTANCE** We utilize a multi-omics approach to demonstrate that diets supplemented with oats, a rich source of microbiota-accessible carbohydrates, are able to confer protection against antibiotic-induced dysbiosis (AID). Our findings affirm that not only is host diet important in shaping antibiotics effects on gut microbiome composition and function but also that the timing of these diets may play an even greater role in managing AID. This work provides a nuanced perspective on dietary intervention against AID and may be informative on preventing AID during routine antibiotic treatment.

## INTRODUCTION

The human gut microbiome is an intricate community comprising bacteria, archaea, fungi, and viruses that reside within the digestive tract. Bacteria represent the best studied members of that community (1). Often, community bacteria mutually benefit their human host by metabolizing host-indigestible fiber, synthesizing essential vitamins, and metabolizing steroidal compounds (2, 3). Host well-being is often tied to gut eubiosis, or stable equilibration and composition, of such bacteria. Gut eubiosis broadly impacts intestinal epithelium maintenance, pathogen colonization resistance, host immunity modulation and training, and metabolism of host-indigestible metabolites (4, 5). Lifestyle and health changes are known to alter the human gut microbiome overtime (2, 6). However, abrupt, rapid, or broad changes that upend or disequilibrate stable communities, termed dysbiosis, contribute to myriad short and long-term morbidities, including metabolic syndromes, autoimmune diseases, and cognitive disorders (7–9).

Dysbiosis is exemplified by pathogenic microbiota overgrowth, decreases in beneficial microbiota, or an overall decrease in microbial diversity. Contributing factors to dysbiosis include individual genetic predispositions, lifestyles, and disease states, but environmental factors like antibiotics and diet are also important determinants (7). Antibiotics are able to rapidly reduce bacterial loads and diversity in the gut microbiome and may lead to antibiotic-induced dysbiosis (AID) (10). Such transient perturbations reduce colonization resistance and increase the risk of infection by pathogens like Clostridioides difficile (11). Similarly, host diet may also change gut microbiota diversity by altering macronutrient availability. For example, “Westernized” diets, high in fat and simple sugars, are often associated with both acute and chronic morbidities (12–15). Such diets reduce gut microbiome diversity, likely by lowering metabolite availability and diversity, especially microbiota-accessible carbohydrates (MACs) like fiber (16). High MAC diets generally increase and maintain gut microbial diversity (16–18). However, previous work suggests that the source and quantity of MACs plays an important role in gut microbiome resilience (19). Dietary microbe-driven changes to gut microbial diversity may significantly alter host physiology, like disrupting host intestinal barrier integrity, which may prompt intestinal inflammation, instigate carcinogenesis, and promote gastrointestinal infections (20, 21). While both antibiotics and diet are powerful modulators of microbial homeostasis, their intersection is poorly defined.

The correlation between microbial growth kinetics and the bactericidal capacity of antibiotics has been appreciated for decades (22, 23). Recent work implies a general link between the metabolic activity of bacteria and antimicrobial sensitivity. Principally, highly metabolically active bacteria are much more susceptible to antibiotic targeting and elimination than less metabolically active bacteria (24–27). Prior work has also suggested that antibiotic exposure is capable of altering both gut microbiome composition and its metabolic activity (10, 28). Prior work tied simple sugar diets to gut microbial community alteration in mice (10, 28) and identified reduced perturbation in polysaccharide supplemented conditions (29). These observations support the notion that diet can modulate the gut microbiome’s response to antibiotics, promoting gut metabolism as a potential target to alleviate or prevent dysbiosis.

While diet is thought to influence the response of the gut microbiome to antibiotics, mobilizing diet to mitigate dysbiosis remains poorly explored. Thus, we sought to determine if a natural source of MACs, oats, could be leveraged temporally to manage AID in the murine gut microbiome. Addressing this, we fed mice diets supplemented with dextrose or milled oats before, during, and after amoxicillin treatment. The impact of oats against murine gut microbiome AID was then assessed using metagenomics and metatranscriptomics. We observe that feeding mice oats before, during, or after treatment with amoxicillin facilitates gut community recovery. We also find that use of oats “prophylactically,” during antibiotic treatment, dramatically mitigates murine gut microbiome AID. This attenuation may be attributed to unique transcriptional changes observed in the gut microbiome of the mice fed oats. This work provides insight into how a tailored diet might be used alongside antibiotics to ameliorate off-target effects during treatment and potentially prevent incidental morbidities.

## RESULTS

### Oats protects taxonomic diversity during amoxicillin challenge and promote recovery.

**FIG 1:**
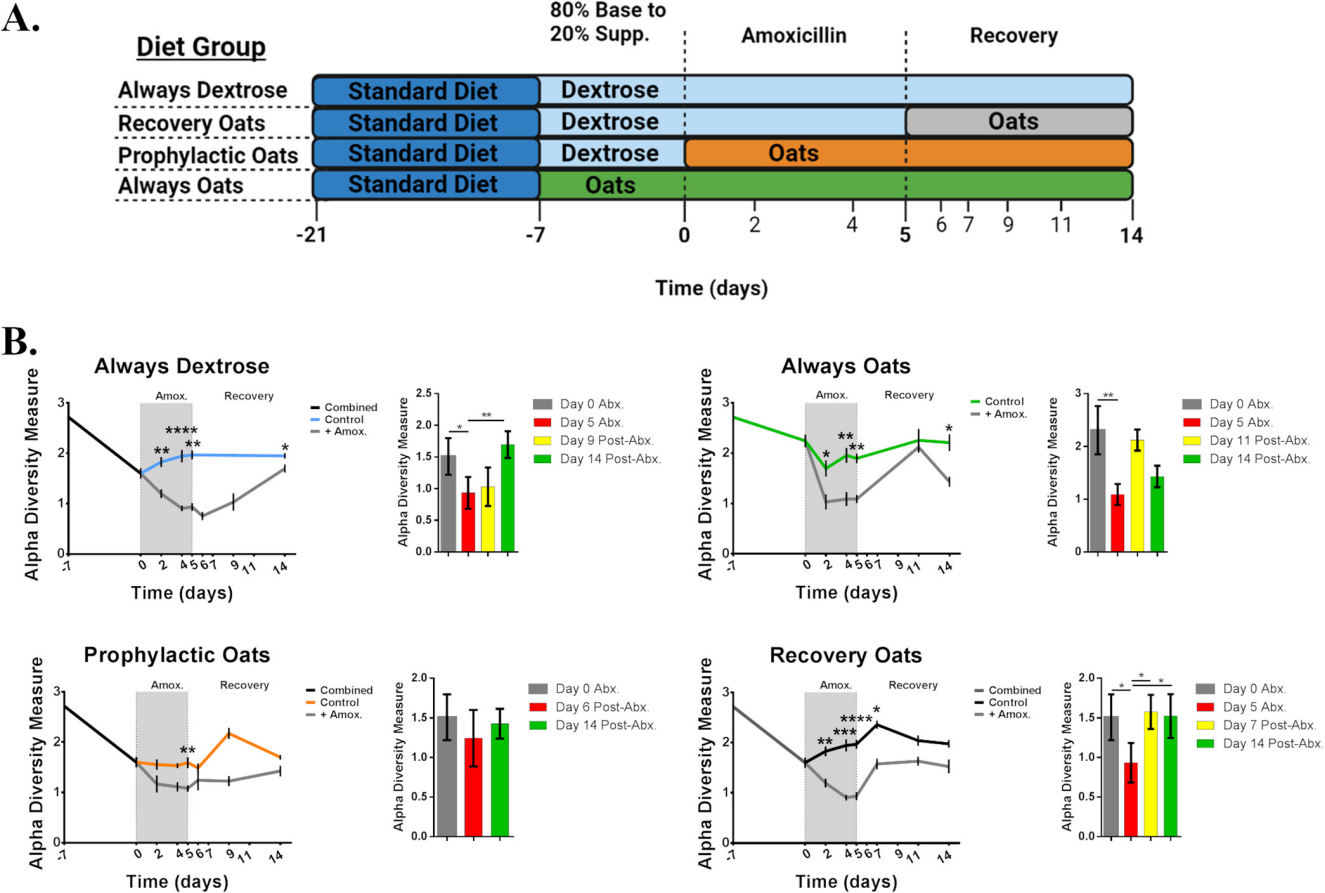
Amoxicillin-induced drops in species diversity are mitigated and subsequent diversity recovery augmented by whole milled oats. (A) Experimental framework used throughout study. Mice are fed a standard diet (LD01; LabDiet, St. Louis, MO, USA) for a 2-week habituation period after which they are randomly subgrouped into the four diet groups and switched to diets comprised of 80% (wt/wt) modified AIN-93G (TD.180901) base diet with 20% (wt/wt) of either pure dextrose or whole milled oats on days −7, 0, or 5 according to the schematic. Amoxicillin challenge group mice in each diet group were administered amoxicillin on day 0 to day 5 and allowed to recover from amoxicillin challenge from end of day 5 to day 14. Fecal pellet samples were collected on days −7, 0, 2, 4, 5, 6, 7, 9, 11, and 14, and cecum samples were obtained on days 0, 5, and 14. (B) Species alpha diversity traces for each diet group along with bar graphs representing species recovery in the amoxicillin challenge groups (always dextrose *n* without amoxicillin for day −7 = 19, day 0 = 9, day 2 = 6, day 4 = 6, day 5 = 10, and day 14 = 4 and with amoxicillin for day 2 = 9, day 4 = 9, day 5 = 10, day 6 = 3, day 9 = 3, and day 14 = 7; recovery oats *n* without amoxicillin for day −7 = 19, day 0 = 9, day 2 = 6, day 4 = 6, day 5 = 10, day 7 = 4, day 11 = 4, and day 14 = 4 and with amoxicillin for day 2 = 9, day 4 = 9, day 5 = 10, day 7 = 4, day 11 = 4, and day 14 = 4; prophylactic oats *n* without amoxicillin for day −7 = 19, day 0 = 9, day 2 = 6, day 4 = 6, day 5 = 3, day 6 = 3, day 9 = 4, and day 14 = 3 and with amoxicillin for day 2 = 6, day 4 = 6, day 5 = 3, day 6 = 3, day 9 = 3, and day 14 = 3; always oats *n* without amoxicillin for day −7 = 6, day 0 = 12, day 2 = 6, day 4 = 6, day 5 = 6, day 11 = 3, and day 14 = 4 and with amoxicillin for day 2 = 6, day 4 = 6, day 5 = 6, day 11 = 3, and day 14 = 4). Traces and bar graphs represent mean sample values and standard error of the mean (SEM) with significance between control and challenge conditions on the same day or between days determined by Mann-Whitney test (*, *P* < 0.05; **, *P* < 0.01; ***, *P* < 0.001; ****, *P* < 0.0001).

To understand how oats alters the gut microbiome under amoxicillin challenge, we conducted the experiment outlined in Fig. 1A. Mice were randomly assigned into the following diet trials: dextrose (always dextrose), whole milled oats (always oats), and transitioning to an oats diet from dextrose during (prophylactic oats) or after (recovery oats) challenge with amoxicillin. Fecal pellet samples were collected throughout the experiment, sequenced for 16S rRNA genes, and these sequences were then used to assess changes in diversity. Alpha diversity drops in each diet group from day −7 to 0, reflecting the gut microbiome steady state transition from the habituation standard chow to the experimental diets (Fig. 1B). The drop in dextrose diet alpha diversity is greater than that of the oats diet condition, reflecting the lower availability of polysaccharides in the dextrose diet. Alpha diversity significantly dropped in each group versus controls at day 2 except for the prophylactic oats group (Fig. 1B). By conducting a simple MIC experiment with Escherichia coli grown with and without oat supplementation, we did not find that amoxicillin susceptibility was directly impacted by the presence of powdered oats. This indicates that the observed attenuation in the host may not be the result of a direct blocking of drug activity (see Table S1 in the supplemental material). Diversity continued to fall after day 2 only in the always dextrose and recovery oats groups, while the prophylactic oats group displayed a significant drop only on day 5 (Fig. 1B). Diversity recovery to day 0 pre-amoxicillin challenge levels occurred as early as day 9 for always dextrose, 11 for always oats, 6 for prophylactic oats, and 7 for recovery oats groups (Fig. 1B). Interestingly, prophylactic oats group diversity levels dropped significantly only on the final day of amoxicillin challenge (see Fig. S1 in the supplemental material). These results suggest transitioning from a simple carbohydrate diet to one high in natural MACs concurrent with or after antibiotic challenge mitigates murine gut microbiome perturbation and facilitates taxonomical recovery.

### Oat-dependent protection from AID is dependent upon timing of supplementation.

**FIG 2:**
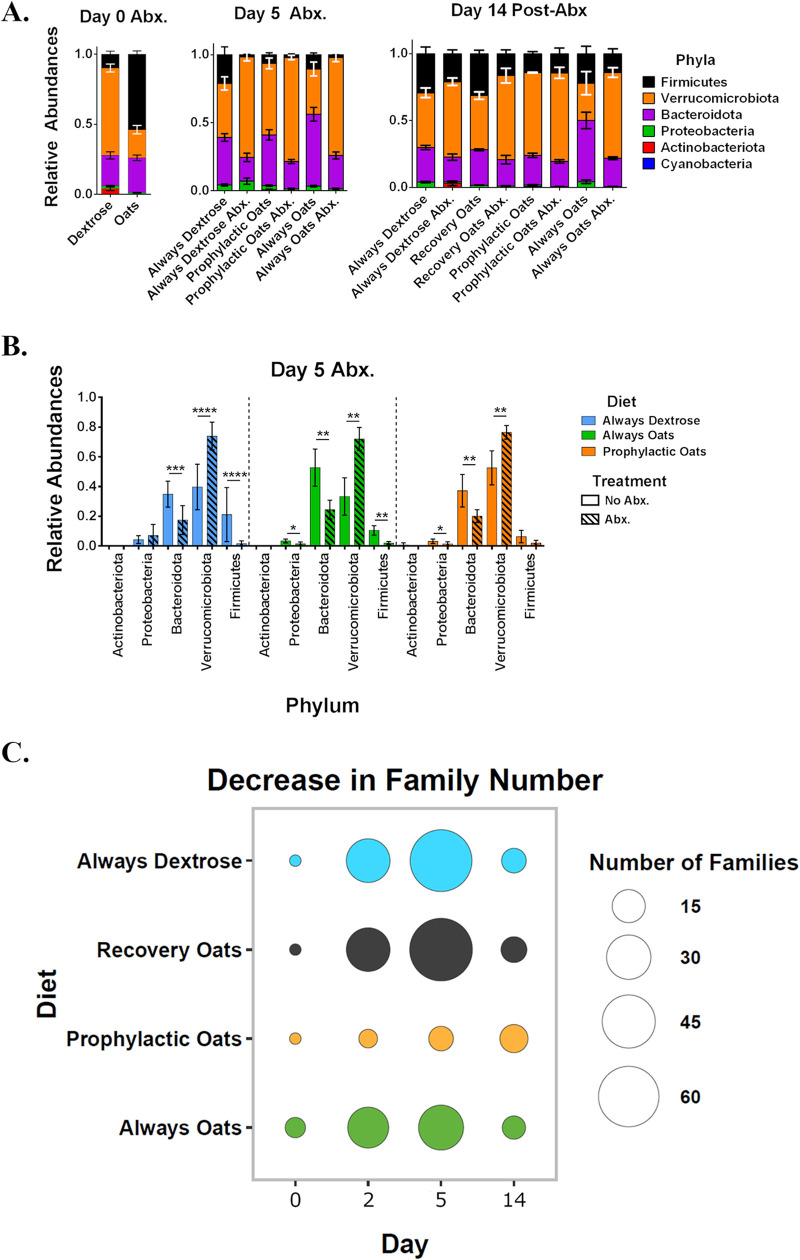
Amoxicillin-induced gut microbiome perturbation is altered by dietary intervention with whole milled oats. (A) Relative abundances of bacterial phyla shown for days 0, 5, and 14 for control and amoxicillin challenged diet groups (always dextrose *n* without amoxicillin for day 0 = 9, day 5 = 10, day 14 = 4 and with amoxicillin for day 5 = 10 and day 14 = 7; recovery oats *n* without amoxicillin for day 0 = 9, day 5 = 10, and day 14 = 4 and with amoxicillin for day 5 = 10 and day 14 = 4; prophylactic oats *n* without amoxicillin for day 0 = 9, day 5 = 3, and day 14 = 3 and with amoxicillin for day 5 = 3 and day 14 = 3; always oats *n* without amoxicillin for day 0 = 12, day 5 = 6, and day 14 = 4 and with amoxicillin for day 5 = 6 and day 14 = 4). (B) Bar graphs representing statistical comparisons of relative abundance data between amoxicillin challenge and control groups for phyla of each diet group. (C) Bubble plot for total number of taxa on the family taxonomical level that decrease under amoxicillin challenge in each diet group over days 0, 2, 5, and 14 as determined by differential abundance analysis with DESeq2. Relative abundance graphs and bar graphs represent mean sample values and SEM with significance between control and challenge conditions of phyla determined by Mann-Whitney test (*, *P* < 0.05; **, *P* < 0.01; ***, *P* < 0.001; ****, *P* < 0.0001).

To elucidate how murine gut community populations respond to amoxicillin between the different diet groups, 16S rRNA sequences were used to derive group population structures. In the dextrose group, *Verrucomicrobiota* compose the community’s majority at day 0 with *Proteobacteria* also present (Fig. 2A), consistent with prior observations (10, 28). *Firmicutes* dominate the population composition in the oats group at day 0 (Fig. 2A). By day 5, all challenged groups indicate *Verrucomicrobiota* as the most abundant taxa, followed by *Bacteroidota* (Fig. 2A). At this time point, *Firmicutes* were the third most abundant phyla in the prophylactic oats and always oats groups while *Proteobacteria* were the third in the always dextrose group (Fig. 2A). By day 14, all of the conditions appear to stabilize toward a similar community composition (Fig. 2A). At this time point, the always oats control group differed, with *Bacteroidota* making up a larger relative share of its community.

To clarify amoxicillin-induced effects on phyla, statistical comparisons were drawn against each phylum within each group (Fig. 2B). Across each group on day 5, the relative proportion of *Bacteroidota* receded and that of *Verrucomicrobiota* increased compared to controls (Fig. 2B). *Proteobacteria* remained unchanged in the always dextrose group but decreased in the always oats and prophylactic oats groups relative to controls (Fig. 2B). *Firmicutes* receded in the always dextrose and always oats diets but did not change significantly in the prophylactic oats group (Fig. 2B). *Actinobacteria* were present at low abundance in the always dextrose and prophylactic oats control groups. For finer taxonomical resolution, family level differential abundance analyses were drawn using DESeq2 (30). By day 5, the always dextrose/recovery oats group had doubled the number of families decreasing at day 2 before returning to pre-amoxicillin levels by day 14 (Fig. 2C). At day 5, the majority of decreasing families were *Firmicutes* (see Data Set S1 in the supplemental material). The always oats group had a similar number of families decreasing on day 2 as the always dextrose/recovery oats group that did not greatly increase by day 5 before returning to pre-amoxicillin levels by day 14 (Fig. 2C). Again, most of the decreasing families on day 5 were *Firmicutes* (Data Set S1). Strikingly, the prophylactic oats diet group had consistently fewer families decreasing on any given day (Fig. 2C). Information on increasing families can be found in Fig. S2 and Data Set S1 in the supplemental material. These results suggest that gut communities provided simple carbohydrate diets are more susceptible to AID than those provided complex carbohydrate diets and show that prophylactic supplementation of oats provides the greatest resistance to perturbation.

### Oats mitigates *Firmicutes* reduction during amoxicillin treatment.

**FIG 3:**
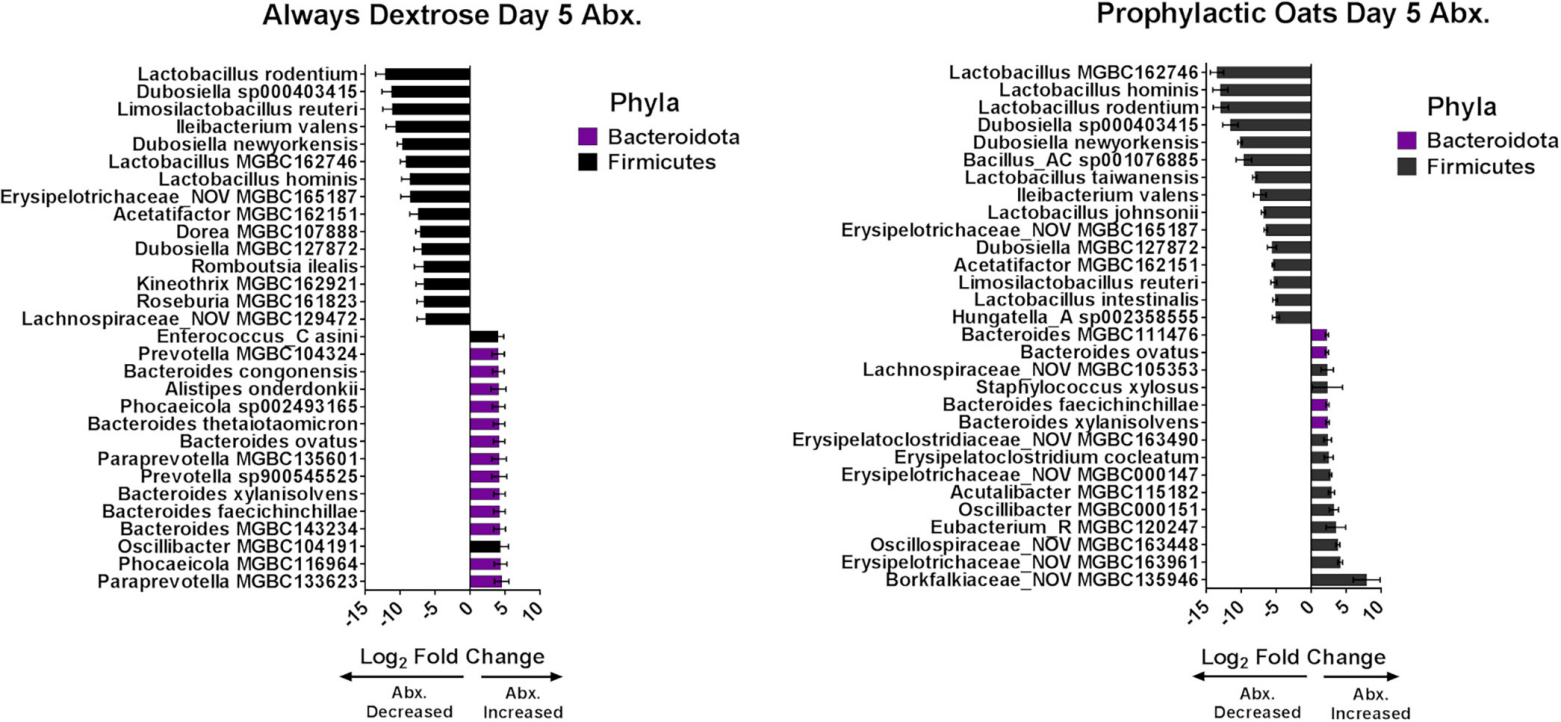
Whole milled oats mitigate amoxicillin-induced drop in species belonging to the *Firmicutes* phylum. Murine cecal content-derived metagenomic reads from the always dextrose and prophylactic oats diet groups are classified against the Mouse Gastrointestinal Bacteria Catalogue (MGBC) premade database (31) with significantly changing species assessed by DESeq2. Results are postprocessed to exclude species with a base mean of <100, a log_2_ fold change of <2, and a *P* value of >0.05. Bars represent mean log_2_ fold change and error bars represent log fold change standard error (*n* = 4).

Since we observed the greatest protective effect with the prophylactic oats diet group, we conducted shotgun metagenomic sequencing of the always dextrose and prophylactic oats groups at the day 5 terminal time point. Here, we utilized cecal material as opposed to fecal samples to generate a more reliable and timely taxonomic and transcriptional profile and then classified reads against the Mouse Gastrointestinal Bacteria Catalogue (MGBC) database (31). Significantly changing species were identified using DESeq2 with results postprocessed as done previously (28). The always dextrose group had nearly 3 times as many species changing significantly than the prophylactic oats group (see Data Set S2 in the supplemental material). Complete DESeq2 results are found in Data Set S3 in the supplemental material. The majority of the always dextrose group increasing species were *Bacteroidota*, while almost a complete majority of the decreasing species were *Firmicutes*. The prophylactic oats group saw significant increases and decreases in species belonging to the *Firmicutes* but fewer were changing in total than in the always dextrose group. The 30 most differentially abundant species are represented in Fig. 3. Corroborating Data Set S2, the majority of decreasing species in either diet group were *Firmicutes*. Likewise, the majority of species increasing in the always dextrose group were *Bacteroidota* and in the prophylactic oats group were *Firmicutes*. Interestingly, Oscillospiraceae_NOV_MGBC163448, Eubacterium_R_MGBC120247, Acutalibacter_MGBC115182, Erysipelotrichaceae_NOV_MGBC000147, and Lachnospiraceae_NOV_MGBC105353 in Fig. 3 were all species found to increase only in the prophylactic oats group. One species, Erysipelotrichaceae_NOV_MGBC163961, was observed to increase in the prophylactic oats group but decrease in the always dextrose group (Fig. 3; see also Data Set S2). These results indicate fewer gut community members change significantly, especially *Firmicutes* members, when fed the oats diet versus the dextrose diet.

### Prophylactic oats elicits a unique functional response from the murine gut microbiome.

**FIG 4:**
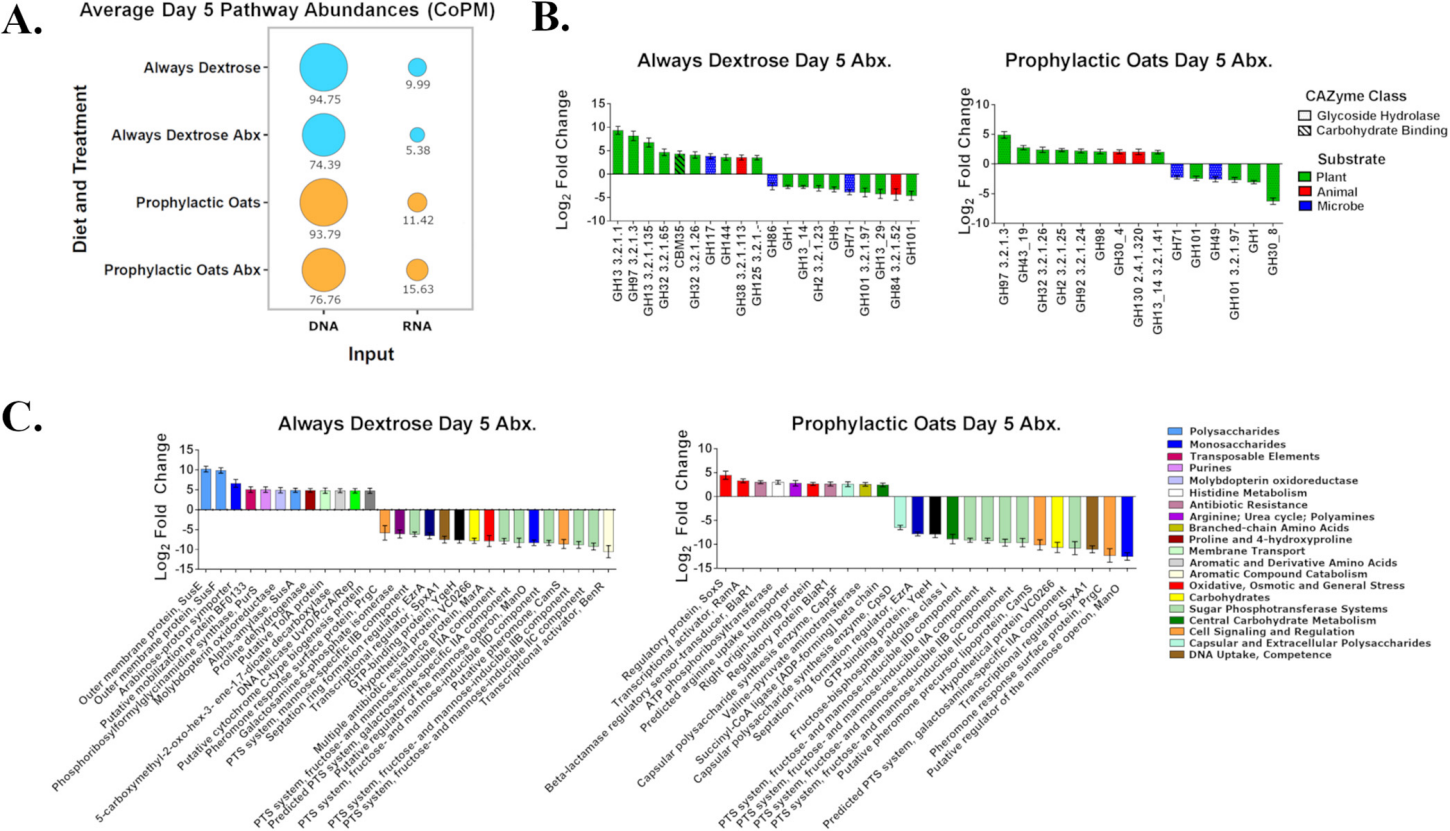
Prophylactic dietary intervention with whole milled oats elicits a unique murine gut microbiome functional response during amoxicillin challenge. (A) Average copies per million (CoPM) pathway abundances for the top HUMAnN3 total expression pathways from metagenomic sequence input (DNA) and metatranscriptomic sequence input (RNA) between the always dextrose and prophylactic oats diet groups for day 5. Aligned reads are normalized to sequence coverage and reported as CoPM. Standard deviation for each condition is as follows: always dextrose DNA, 45.93, and RNA, 8.56; always dextrose Abx DNA, 52.23, and RNA, 4.44; prophylactic oats DNA, 41.88, and RNA, 9.03; and prophylactic oats Abx DNA, 34.67, and RNA, 8.82. (B) Differentially expressed CAZyme transcripts from murine cecal metatranscriptome in mice consuming either the always dextrose or prophylactic oats diets on day 5 of amoxicillin challenge. Bars represent mean log_2_ fold change, and error bars represent log fold change standard error (*n* = 4). Substrate legend indicates the primary source of the target substrate for the associated CAZyme. (C) Differentially expressed SEED transcripts at level 4 from murine cecal metatranscriptome in mice consuming either the always dextrose or prophylactic oats diets on day 5 of amoxicillin challenge. Bars represent mean log_2_ fold change, and error bars represent log fold change standard error (*n* = 4). Level 1 SEED designation along with color scheme is indicated below the graph.

We assessed how the two diet group gut communities respond functionally to amoxicillin challenge using cecal metatranscriptomics with classification against MetaCyc and a custom protein MGBC database. Average abundance patterns of the top pathways between the groups are reported in copies per million (CoPM) in Fig. 4A. The heat map corresponding to top pathways may be found in Fig. S3 in the supplemental material, and complete data sets can be found in Data Sets S4a and S4b in the supplemental material. While metagenomic pathways (DNA) between the two diet groups and their treatment conditions were comparable, we note that metatranscriptomic activity (RNA) of the amoxicillin-treated prophylactic oats group was nearly 3 times greater than amoxicillin-treated always dextrose group (Fig. 4A). We also assessed how the diets alter carbohydrate utilization relative to amoxicillin challenge by aligning reads to the carbohydrate-active enzymes (CAZy) database (32). The complete list of CAZyme hits are found in Data Set S5 in the supplemental material. We found that the majority of increasing CAZymes in the always dextrose group involved mucus glycoprotein carbohydrate use and glucose acquisition from carbohydrate polymers (Fig. 4B; see also Data Set S5). The majority of significantly decreasing CAZymes related to complex polysaccharide use save for glycogen synthase (GT5). All of the increasing prophylactic oats CAZymes were involved in complex polysaccharide utilization (Fig. 4B). Those decreasing significantly involved catabolism of simpler complex carbohydrate polymers or the acquisition and utilization of glycoprotein carbohydrates (Fig. 4B).

Broad functional differences between the diet groups were then investigated by aligning metatranscriptomic reads to the SEED subsystems database (33). We found that the Always Dextrose group had more than 3 times the number of significantly changing subsystems than the prophylactic oats group (see Data Set S6 in the supplemental material). Of the subsystems increasing only in the always dextrose group, most related to anaerobic respiration, complex or simple carbohydrate sourcing, starch utilization, the tricarboxylic acid (TCA) cycle, the pentose phosphate pathway (PPP), and oxidative stress resistance. Those decreasing involved complex carbohydrate utilization, phosphotransferase system (PTS) simple sugar import, fermentation-related pathways, and glycogen synthesis (Fig. 4C; see also Data Set S6), with subsystems increasing only in the prophylactic oats group related to anaerobic metabolism, oxidative or general stress, and antibiotic resistance pathways. Those decreasing involved NAD^+^ cofactor regeneration, the PhoB phosphate regulon, acetamido biosynthesis, and two PTS member’s for either maltose/glucose or sucrose transport (Fig. 4C; see also Data Set S6). Altogether, these results suggest that the always dextrose gut communities emphasize carbohydrate sourcing, respiratory metabolism, and oxidative stress management, while the prophylactic oats communities shift metabolism and favor generalized stress and antibiotic management.

### The gut microbial community stress response profile is altered by oats.

**FIG 5:**
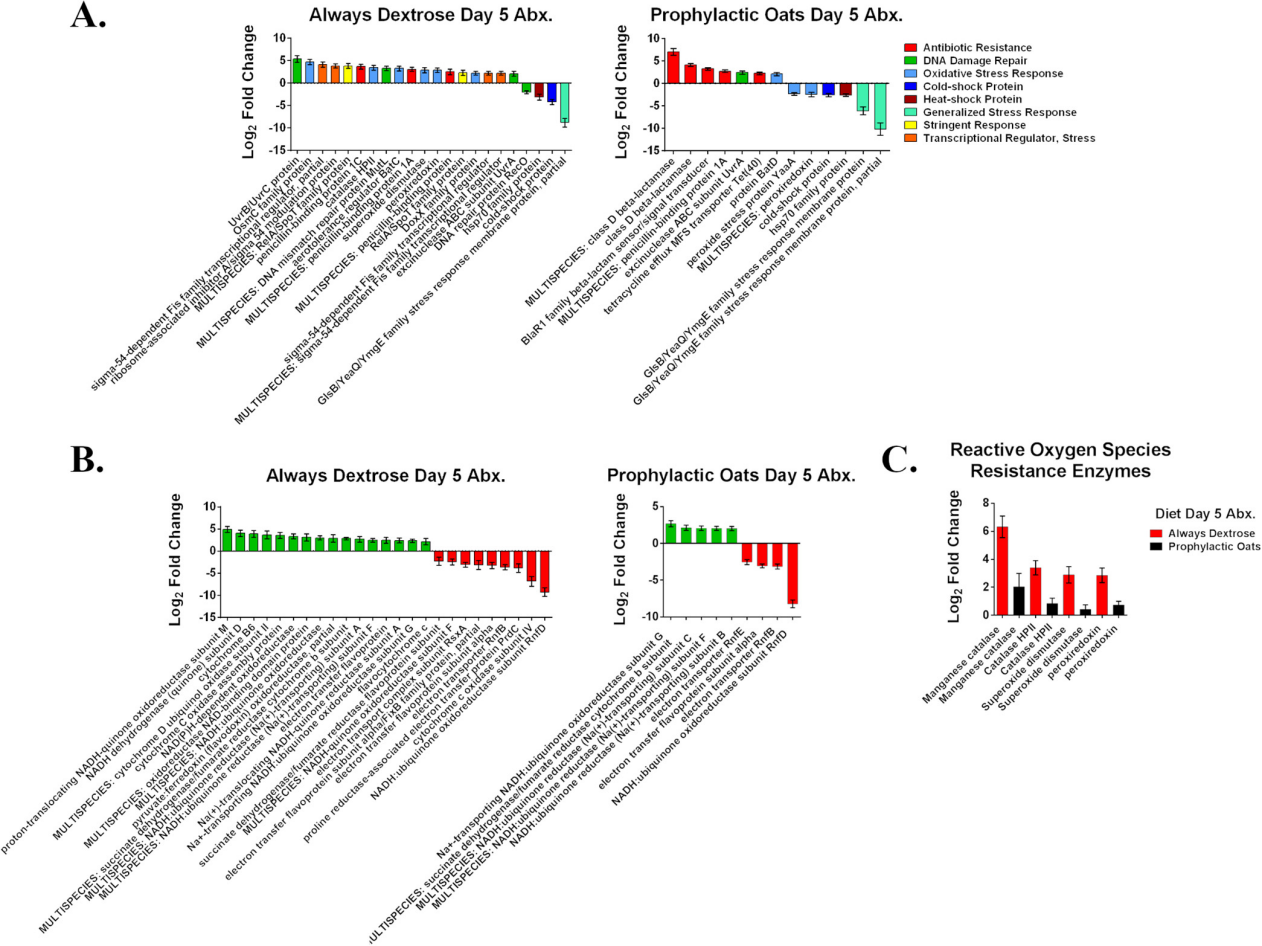
Whole milled oats alter the gut microbial community stress response profile. (A) Differentially expressed RefSeq transcripts from murine cecal metatranscriptome in mice consuming either the always dextrose or prophylactic oats diets on day 5 of amoxicillin challenge. The legend indicates the family of functional roles each feature is associated with. (B) Differentially expressed RefSeq transcripts relating to electron transport chain proteins for the always dextrose and prophylactic oats diet groups on day 5 of amoxicillin challenge. (C) Differentially expressed RefSeq transcripts relating to reactive oxygen species resistance enzymes in the always dextrose and prophylactic oats diet groups on day 5 of amoxicillin challenge. Each figure bar represents mean log_2_ fold change and error bars represent log fold change standard error (*n* = 4).

To further characterize the stress management profiles of the target diet groups, we aligned metatranscriptomic reads to the Reference Sequence (RefSeq) (34) database. The complete list of hits can be found in Data Set S7 in the supplemental material. We found that the always dextrose group had greater than 3 times the number of total RefSeq features changing significantly than the prophylactic oats group (Data Set S7). From these total pools, all stress resistance/management genes and features were identified and visualized in Fig. 5A. The increasing always dextrose group features involved oxidative stress resistance/management and general stress management with those decreasing largely related to general stress response features (Fig. 5A). The increasing prophylactic oats group features heavily skewed toward antibiotic resistance while those decreasing were similar to the always dextrose group features except for additional oxidative stress resistance features (Fig. 5A). We next searched the feature list for electron transport chain proteins to gauge gut community commitment to respiratory energy production. We found 25 such features changing significantly in the always dextrose group and 9 in the prophylactic oats group (Fig. 5B). We then assessed the feature list to determine if markers of oxidative stress management were higher in the always dextrose versus prophylactic oats group. Of the 4 oxidative stress mitigation markers found, all were elevated higher in the always dextrose group than the prophylactic oats group (Fig. 5C). These results imply the always dextrose gut community is committed more to managing metabolic stress than managing antibiotic resistance like in the prophylactic oats community.

### Oats alters Bacteroides thetaiotaomicron and Akkermansia muciniphila gene expression under amoxicillin challenge.

**FIG 6:**
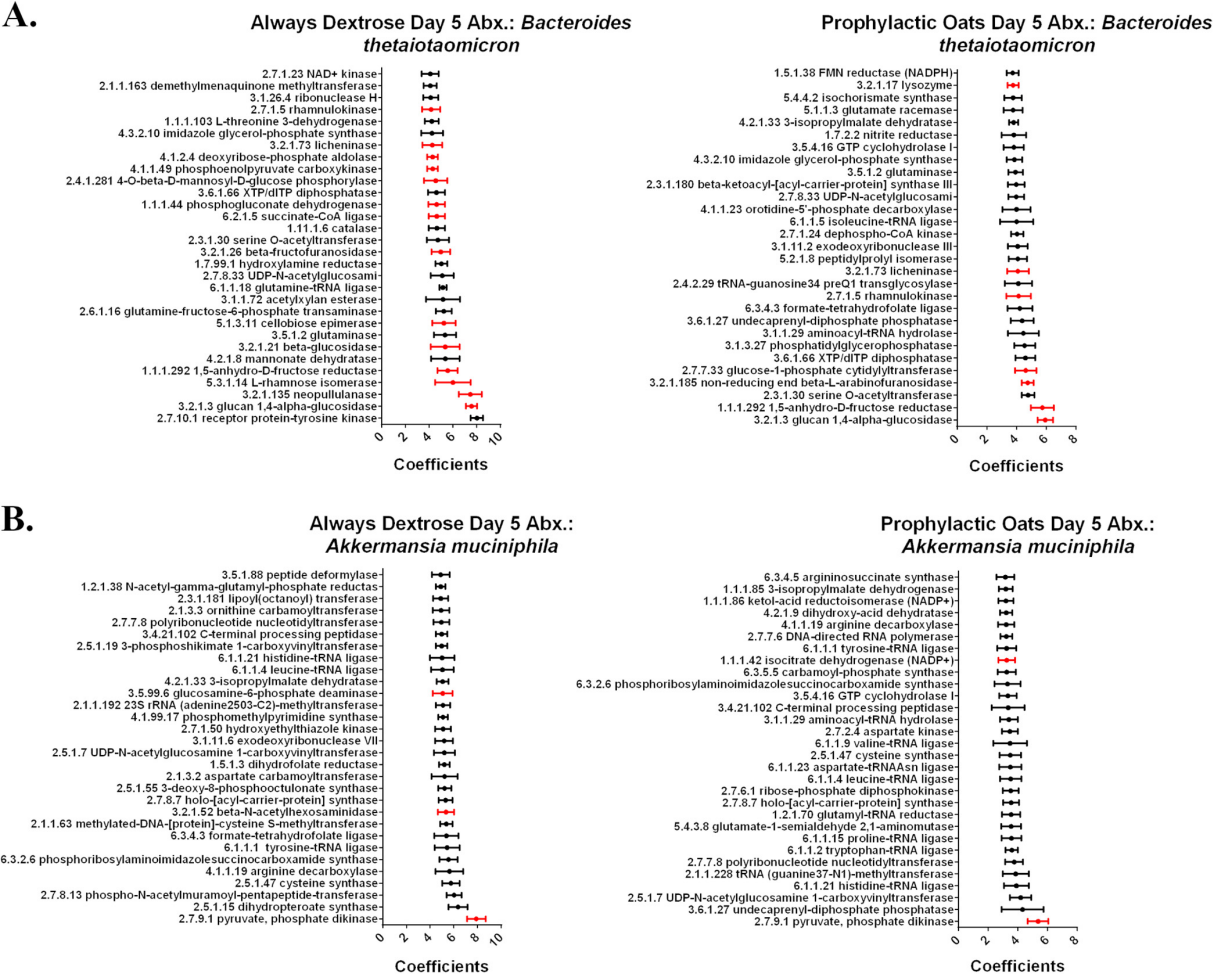
Bacteroides thetaiotaomicron and Akkermansia muciniphila alter gene expression during amoxicillin challenge under whole milled oats dietary intervention. (A) The top 30 features, by greatest effect size, of microbiome multivariate association with linear models (MaAsLin2) (42) differential abundance analyzed HUMAnN3 species level functional information for B. thetaiotaomicron. (B) The top 30 features, by greatest effect size, of MaAsLin2 differential abundance analyzed HUMAnN3 species level functional information for *A. muciniphila*. Features highlighted in red relate to glycolysis/gluconeogenesis, the tricarboxylic acid cycle, the pentose phosphate pathway, and simple and complex carbohydrate metabolism. Each feature is represented as the model coefficient value (effect size) with model standard error for an *n* = 4.

To characterize diet influence on individual microbial responses to amoxicillin, we assessed the transcriptional profiles of two abundant gut commensals: B. thetaiotaomicron and *A. muciniphila*. Species functional information derived from HUMAnN3 metatranscriptomic outputs were analyzed by microbiome multivariable association with linear models (MaAsLin2) (35) with the top 30 features by effect size represented in Fig. 6. The complete list of all species features can be found in Data Set S8 in the supplemental material. All features involving glycolysis/gluconeogenesis, the TCA, the PPP, and simple and complex carbohydrate metabolism for the two species were then identified in Data Set S8. In the top 30 B. thetaiotaomicron features, nearly half of the always dextrose condition features related to the targeted metabolic features while fewer were observed in the prophylactic oats condition (Fig. 6A). Interestingly, lysozyme, known prior to improve composition and metabolic function of sow gut microbiota (36), was present only in the prophylactic oats group (Fig. 6A; see also Data Set S8). In the top 30 *A. muciniphila* features, comparably few targeted metabolic features were identified in either diet condition (Fig. 6B). However, β-*N*-acetylhexosaminidase, an enzyme that catalyzes the cleavage of β-*N*-acetylglucosamine residues from glycoproteins (37), was present only in the always dextrose group (Fig. 6B; see also Data Set S8). A shared target feature comparison for the two species and diet groups is found in Fig. S4 in the supplemental material. These results indicate a shift in response to diet and amoxicillin challenge that corroborates our prior results from the full community analyses and provides further evidence for metabolic-driven AID tolerance.

## DISCUSSION

Host diet is known to modulate antibiotic-induced gut microbiome disruption largely within the context of microbial metabolism (10, 28, 29). This relationship is exemplified by organisms like Mycobacterium tuberculosis whose infectious lifestyle is marked by slow growth, linking metabolism to antibiotic efficacy (38–41). In essence, bacteria actively metabolizing are more susceptible to antibiotic elimination through energy expensive futile cycling and deleterious metabolic by-product accumulation processes than less metabolically active bacteria (24–27, 42–44). Consequently, diets poor in complex microbially accessible carbohydrates and rich in simple carbohydrates and fats are often linked to dysbiotic guts and precipitate acute and chronic morbidities (12–15, 17). While modulating diet and thus microbe nutrient availability are major contributors to antibiotic susceptibility (10, 24–26, 44–50), much remains unclear regarding the how, when, in what form, and in what quantity to leverage dietary therapeutics against AID (19).

Here, we used a multi-omics approach to demonstrate that an oat diet, replete in MACs, is able to modulate the murine gut microbiome response to amoxicillin in a temporal fashion. Through 16S rRNA analysis, we determined that phylum-level community structures changed comparably between the different diet groups throughout the experiment. However, the changes to the overall prophylactic oats diet group community were reflective of far fewer actual microbial species changing than in any other diet group. We also observed a protective effect on *Firmicutes*, whose members are often among the first bacteria to utilize MACs (51), against amoxicillin in the prophylactic oat diet compared to that in the dextrose diet. Furthermore, *Proteobacteria*, often cited clinically as major human disease agents (52), were found to significantly decrease in the oat diets after amoxicillin treatment but remained unchanged in the always dextrose diet. This illustrates that while oats are able to mitigate AID better than a diet comprised of dextrose, what mattered most to this effect was when the dietary transition to oats occurred. Pursuing this temporal aspect further, our metatranscriptomic results demonstrated that the prophylactic oats group leverages an entirely different response to amoxicillin than the always dextrose group. While the always dextrose diet group heavily emphasized carbohydrate scavenging, amino acid metabolism, and managing metabolic stress, the prophylactic oats diet group favored less energetic metabolic pathways and direct management of antibiotic resistance. This observation affirms metabolic-driven antibiotic resistance but bolsters a second layer to our observed community resilience in the form of direct antibiotic resistance. This clear functional contrast may be due to the overall shift in community composition between the two diets, reflect directly on community metabolic state, or likely encapsulates both. Defining the transcriptional profiles of the two abundant gut commensals, *A. muciniphila* and B. thetaiotaomicron, corroborated our metabolic observations and supported the idea of colonic mucus breakdown in fiber-deprived guts (19). Specifically, *A. muciniphila* in the always dextrose diet group highly elevated glucosamine-6-phosphate deaminase and, especially, β-*N*-acetylhexosaminidase, suggesting mucus glycoprotein metabolism was favored in that diet group. The total elevated metabolic features of B. thetaiotaomicron in the always dextrose diet group indicates a broad emphasis on carbohydrate sourcing and utilization while the prophylactic oats diet group favored complex carbohydrates and linked metabolic pathways. These results taken together imply always dextrose group communities were attempting to scavenge carbohydrates for respiratory metabolic processes while the prophylactic oats group preferred select respiratory and fermentative processes, consistent with prior work (10, 29). Ultimately, this work expands upon the idea that diets high in sugars or other easily host-accessible metabolites over MACs not only starves the gut microbiota of critical resources for proper gut community maintenance but also facilitates and exacerbates AID (17).

Complicating the interpretation of our results are the drawbacks inherent to our experimental design and the multi-omics approach. In our experiments, only female mice were used where currently an appreciation that inherent sex-dependent differences exist, and thus, our observations may not necessarily be generalizable to males (53–55). While we demonstrate amoxicillin function is not directly hindered by oat sequestration (see Table S1 in the supplemental material), the impact of oats on gastrointestinal transit time was not assessed. Evidence does suggest that diet can impact transit time and that transit time can impact drug activity (56). Thus, this factor should be considered as a possibility in future work. However, since both the always oats and prophylactic oats diet contained oats at the time of amoxicillin exposure, the transit time likely does not play a role in the difference between these conditions. Intrinsic drawbacks to our analytical pipeline include the necessary reliance on incomplete databases and the relative nature of our 16S amplicon and shotgun metagenomics approaches. While the metagenomic analysis was conducted on a consistent number of samples, 16S sample sizes varied due to experimental setup and animal husbandry considerations, thus limiting assessment of rare and low-amplitude changes. However, our 16S analysis goal was to broadly assess population scale shifts and not detect rare or marginally shifted taxa; thus, our sample sizes were sufficient to identify such changes. Overall, assigning biological significance in our microbiome analyses is difficult, and we necessarily cannot comment affirmatively on whether taxonomical changes occur due to increases or decreases in special biomass or simply by relative proportional changes occurring within the community as a whole. Similarly, the complexity inherent in whole-system studies makes it challenging to attribute transcriptional changes to direct dietary intervention or indirect changes throughout the system. Our prior work demonstrates that antibiotic treatment alters metabolic by-products concomitantly with transcriptional changes (57). Leveraging metabolomics alongside metatranscriptomics and metagenomics in future work may clarify functional changes occurring during oat dietary intervention. Regardless, this study enhances our understanding of the importance of high MAC diets, especially those from natural sources like oats, while simultaneously highlighting the importance of considering timing in the use of such diets against incidental antibiotic-induced morbidity. Future work should focus more on the temporal dimension of AID dietary intervention using natural sources of MACs. Furthermore, resolving how constituent components of whole milled oats like (1,3;1,4)-β-glucan and phytochemicals contribute toward temporal AID resilience may provide mechanistic insights into oat dietary intervention.

## MATERIALS AND METHODS

### Study animals.

All animal procedures were approved by the Brown University Institutional Animal Care and Use Committee under protocol 20-06-0001. Seventy-two 5-week-old female C57BL/6J mice were obtained from The Jackson Laboratory (Bar Harbor, ME, USA) and housed in Brown University’s specific-pathogen-free Animal Facility.

### Rodent diets.

Mice were fed LabDiet 5001 (LD01; LabDiet, St. Louis, MO, USA) feed pellets for a 2-week habituation period. For experimental diets, a custom base diet reflecting standard mouse chow fiber content was designed (Envigo-Teklad, Madison, WI, USA). The base diet modifies AIN-93G (TD.180901) by removing all cellulose and reducing cornstarch content while adjusting micro- and macronutrient contents so the diet could be used as an 80% base for 20% (wt/wt) supplementation with other carbon sources. BUFFALO cornstarch (Ingredion Incorporated, IL, USA) was used to increase host accessibility and reduce cornstarch availability in the cecum and lower gastrointestinal tract. The dextrose diet was a powdered diet of 80% base and 20% glucose (Fisher Scientific, Waltham, MA, USA). The oats diet was a powdered diet of 80% base diet and 20% whole milled oats. Whole milled oats were prepared by liquid nitrogen snap-freezing and complete milling followed by baking at 120°C for 20 min. Five grams per mouse of irradiated powdered diets were provided to mice daily to consume *ad libitum*.

### Animal experiments.

Following habituation, mice were grouped randomly and then acclimated for 1 week on either the dextrose or oats diet. Mice were then grouped into cages according to continuing a dextrose (always dextrose group) or oats (always oats group) diet or switching from dextrose to oats (prophylactic oats group). Groups where subdivided into control or amoxicillin-treated cages and provided *ad libitum* filter-sterilized water or filter-sterilized water with 0.1667 mg/mL amoxicillin. Treatment cages were provided fresh amoxicillin water bottles daily. Amoxicillin challenge course lasted 5 days. Following treatment, a subgroup of the always dextrose group was switched from the dextrose to the oats diet (recovery oats group). Mice then recovered for 9 days until experiment completion. Fecal samples were collected and stored in DNA/RNA shield (Zymo Research; Irvine, CA, USA) at −80°C on days −7, 0, 2, 4, 5, 6, 7, 9, 11, and 14. Cecum samples were collected in bead-bashing tubes with DNA/RNA shield and stored at −80°C on days 0, 5, and 14.

### DNA and RNA extraction and quantification.

Total DNA and RNA was liberated from bacteria by bead-bashing fecal or cecum samples for 5 min on a Bead Ruptor 96 (Omni International, Kennesaw, GA). Fecal DNA was extracted according to the manufacturer’s instructions using the Fecal/Soil Microbe 96 MagBead kit (D6011-FM, Irvine, CA, USA). Total DNA and RNA were coextracted and isolated from cecum samples according to the manufacturer’s instructions using the ZymoBIOMICS MagBead DNA/RNA kit (R2136). Nucleic acids were eluted in nuclease-free water and quantified using the double-stranded DNA high sensitivity (dsDNA-HS) or RNA-HS kits on a Qubit 3.0 fluorometer (Thermo Fisher Scientific, Waltham, MA, USA).

### 16S rRNA amplicon preparation and sequencing.

The 16S rRNA gene V4 region was amplified from fecal total DNA using barcoded 515F forward and indexed 806R reverse primers as previously (58). Amplicons were generated with Phusion high-fidelity DNA polymerase (F530L; Thermo Fisher) following the program earth microbiome protocol (58). Amplicons were verified by gel electrophoresis, quantified on the Qubit 3.0 and pooled in equimolar concentrations. Amplicons were paired-end (2 × 250 bp) sequenced by Illumina MiSeq sequencing using the 600-cycle kit according to manufacturer’s protocols at the Rhode Island Genomics and Sequencing Center at the University of Rhode Island. The average read depth was 31,737 (±15,392) reads per sample.

### 16S sequence processing and analysis.

16S rRNA gene sequences were demultiplexed with idemp (59), quality filtered, trimmed, and denoised with the DADA2 (60) QIIME 2 plugin (q2-dada2) and then merged with QIIME 2 (version 2022.8) (61). Sequence variants were aligned and a phylogenetic tree derived with MAFFT (62) (q2-alignment) and FastTree 2 (63) (q2-phylogeny). Taxonomic information was assigned through a pretrained naïve Bayes classifier (q2-feature-classifier) (64) trained on the SILVA 132 99% database (65). Taxonomic diversity was assessed by alpha (Shannon & Faith’s phylogenetic diversity) and beta diversity (Bray-Curtis dissimilarity) metrics through the phyloseq package (66, 67) (version 1.38.0) in R (version 4.1.3).

### Metagenomic and metatranscriptomic library preparation.

Metagenomic libraries were generated from 100 ng of DNA using the NEBNext Ultra II FS DNA library prep kit (E7805L; New England BioLabs, Ipswich, MA, USA) with ≥100 ng input protocol per manufacturer’s instructions. Pool fragments ranged from 250 to 1,000 bp averaging to ~400 bp. Metatranscriptomic libraries were prepared with ~1 μg of total RNA using the NEBNext Ultra II directional RNA sequencing prep kit (E7760S) with the NEBNext rRNA depletion kit for human/mouse/rat (E6310L) and the MICROBExpress kit (AM1905; Invitrogen, Carlsbad, CA, USA) according to manufacturer’s instructions. Pool fragments ranged from 200 to 500 bp averaging to ~275 bp. Metagenomic and metatranscriptomic libraries were pair-end sequenced (PE150) on the NovaSeq 6000 (Novogene, Sacramento, CA, USA). Metagenomic samples averaged 35,780,052 (±20,198,450) reads per sample and metatranscriptomic samples averaged 152,707,388 (±33,779,684) reads per sample.

### Metagenomic and metatranscriptomic short-read processing.

Raw metagenomic and metatranscriptomic reads were trimmed and decontaminated with Trimmomatic (68) (version 0.39) and KneadData (69) (version 0.6.1). Trimmomatic removed low-quality reads and Illumina TruSeq3 adapter sequences with the SLIDINGWINDOW value 4:20, ILLUMINACLIP value 2:20:10, and MINLEN value 75. Quality controlled reads were decontaminated using bowtie2 (70) (version 2.2) removing reads mapping to the C57BL/6J mouse genome or two murine retroviruses found in our animal facility: murine mammary tumor virus (MMTV) (GenBank accession number NC_001503) and murine osteosarcoma virus (MOV) (GenBank accession number NC_001506.1). Raw metatranscriptomic reads were processed similarly except for an additional decontamination of sequences aligning with the SILVA 128 LSU and SSU Par crRNA databases (71).

### Metagenomic short-read classification.

Metagenomic reads were classified against the pre-built Mouse Gastrointestinal Bacteria Catalogue (MGBC) Kraken 2/Bracken database (31) using Kraken 2 (version 2.0.7-beta) with default k-mer and l-mer values (72). Phylum and species abundances were calculated with Bracken (73) (version 2.7.0) using Kraken 2 reports.

### Metagenomic and metatranscriptomic analysis with HUMAnN3.

Metagenomic and metatranscriptomic expression changes were identified using HUMAnN3 (74). Reads were classified to bacterial species using a custom pangenome database. The custom database was built using the MGBC genome database (75). Briefly, gene prediction from assembled contigs was achieved using Prodigal (76) (version 2.6.3) with gene duplicates per genome removed using vsearch (77) (version 2.21.1). Genomes were concatenated into a single FASTA file with genes clustered by sequence identity and assigned UniRef90 database (78) annotations with MMseqs (79) (version 13.45111), ultimately generating the final annotated FASTA file. A custom protein database was built using the same process but starting with the amino acid output from Prodigal. Classified reads were aligned to this custom protein database to identify functional expression and to the MetaCyc database (80) to identify expression pathways. Aligned reads are normalized to sequencing coverage and reported as copies per million (CoPM).

### Metatranscriptomic analysis with SAMSA2.

Reads were annotated through a modified version of the Simple Annotation of Metatranscriptomes by Sequence Analysis 2 (81) (SAMSA2) pipeline as previously described (57). Reads are quality controlled and merged with Paired-End Read Merger (82) (PEAR) then aligned against to the CAZyme (32), SEED Subsystem (33), and RefSeq (34) databases with DIAMOND (83) (version 0.9.12).

### Statistical analysis.

Initial output analyses were conducted by linear discriminant analysis effect size (LEfSe) (84) Galaxy web server (Galaxy version 1) under default settings. Kraken 2/Bracken Metagenomic outputs and SAMSA2 metatranscriptomic outputs were tested for differential abundance using the DESeq2 package (30) (version 1.34.0) under default parameters with Benjamini-Hochberg correction. HUMAnN3 metatranscriptomic outputs were tested for differential abundance using the microbiome multivariable association with linear models (MaAsLin2) package (35) (version 1.8.0) under default parameters. Mann-Whitney unpaired *t* tests and Kruskal-Wallis one-way analyses of variance (ANOVAs) were performed in GraphPad Prism (version 6.0). All experiments represent biological replicate data, and details of specific statistical analyses for all experiments are defined in figure legends.

### Data availability.

Data sets used throughout are made available through the NCBI Sequence Read Archive (SRA) under BioProject accession numbers PRJNA992406 (metagenomics and metatranscriptomics) and PRJNA991569 (16S rRNA amplicon sequences). Additional information is available from the corresponding author upon request.
