# Supplementary material for: Short-Term Dietary Intervention with Whole Oats Protects from Antibiotic-Induced Dysbiosis
Source: Microbiol Spectr. 2023 Jul 13;11(4):e02376-23. doi: 10.1128/spectrum.02376-23 (PMC10434222; doi:10.1128/spectrum.02376-23)
Supplement: Supplemental file 10 — Supplemental material. Download spectrum.02376-23-s0001.pdf, PDF file, 0.7 MB [file spectrum.02376-23-s0001.pdf]

Supplementary Table 1. Effect of Dextrin or Whole Milled Oats on Amoxicillin MIC of select *Escherichia coli*

		Strain (MIC ug/mL)	
		MG1655	UMN308 UPEC
	LB	4	4
Dextrin	0.125%	8	8
	0.25%	4	4-8
	0.5%	4	4-8
Whole Milled Oats	0.125%	8	4-8
	0.25%	8	4
	0.5%	8	4

Percent Dextrin or Whole Milled Oats is determined as w/v.
10^6 CFU/mL of each strain was assessed anaerobically at 37˚C.
Amoxicillin sensitive is defined as ≤ 8 ug/mL, intermediate > 8 ug/mL & < 32 ug/mL, resistant breakpoint at 32 ug/mL.
All experiments represent values from 4 biological replicates.

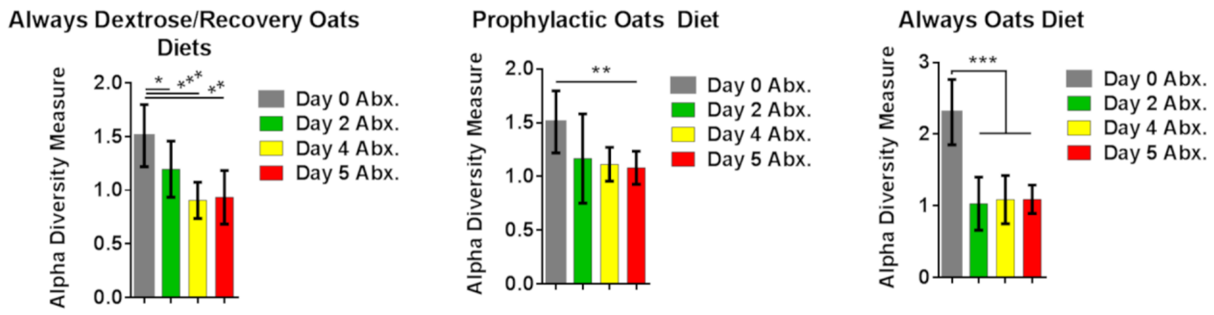

Supplementary Figure 1. Prophylactic Oats diet group resists species diversity drop throughout amoxicillin challenge. Bar graphs representing species diversity drop in the amoxicillin challenge groups for each diet group over days 0, 2, 4 and 5 of amoxicillin challenge (n = 3-10). Graphs represent mean sample values and SEM with significance between days determined by Mann-Whitney test (* = $p<0.05$, ** = $p<0.01$ & *** = $p<0.001$).

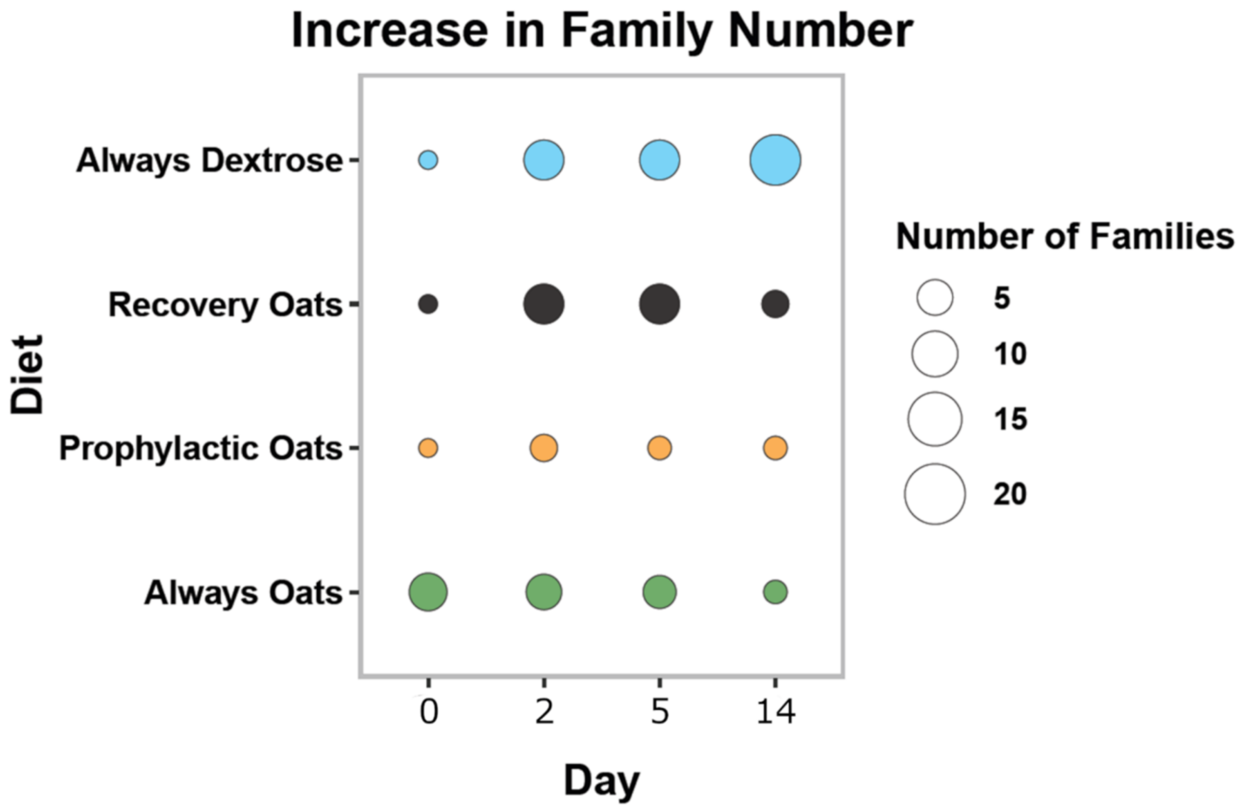

Supplementary Figure 2. Numbers of significantly increasing families in each diet group throughout amoxicillin challenge. Bubble plot for total number of taxa on the family taxonomical level that significantly increase under amoxicillin challenge in each diet group over days 0, 2, 5 and 14 as determined by differential abundance analysis with DESeq2.

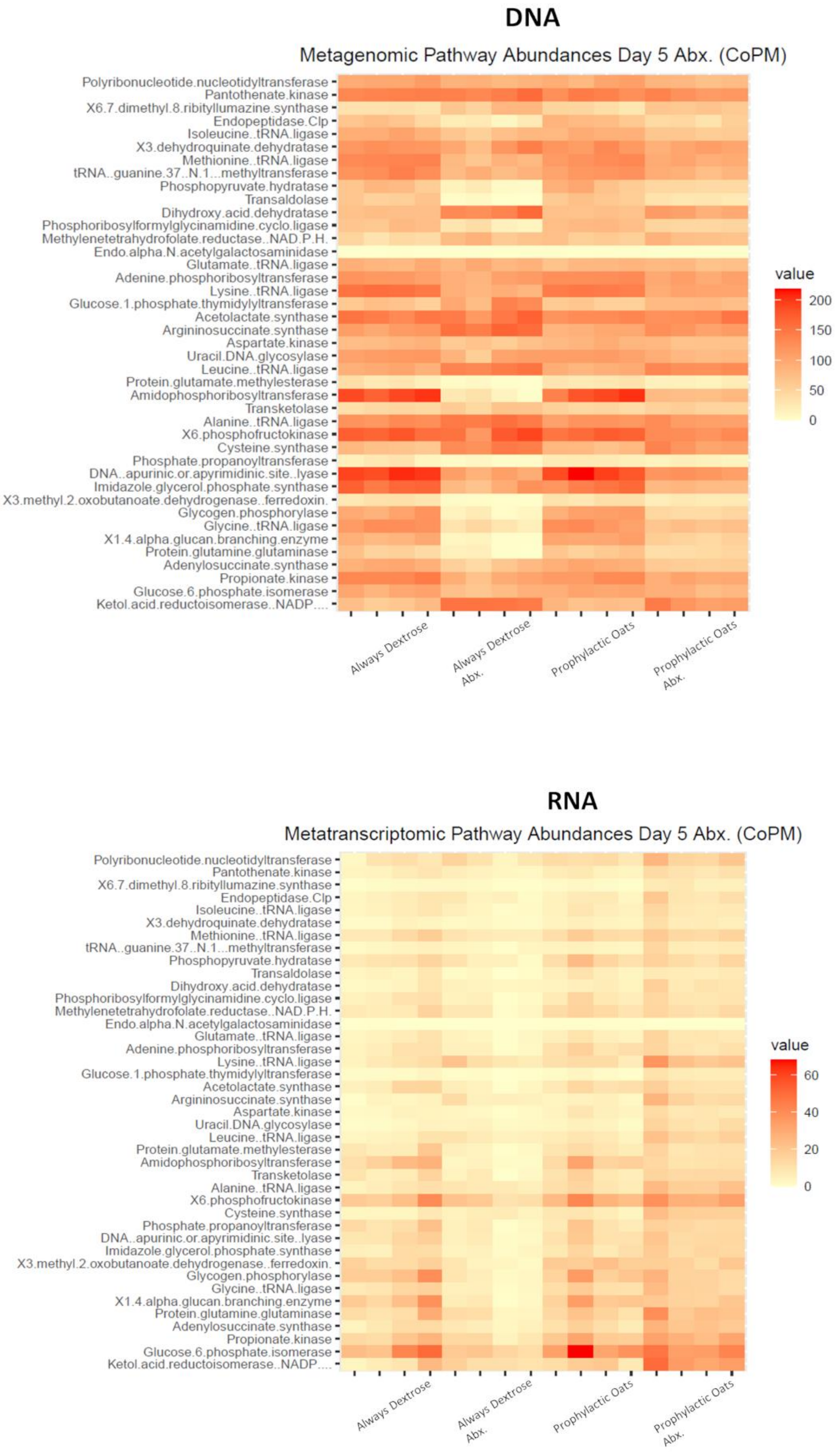

Supplementary Figure 3. HUMAnN3 total expression pathways for top metagenomic and metatranscriptomic sequence input pathways. HUMAnN3 total expression pathways for the top 41 pathways from metagenomic sequence input (DNA) and metatranscriptomic sequence input (RNA) between the Always Dextrose and Prophylactic Oats diet groups for day 5. Aligned reads are normalized to sequence coverage and reported as copies per million (CoPM).

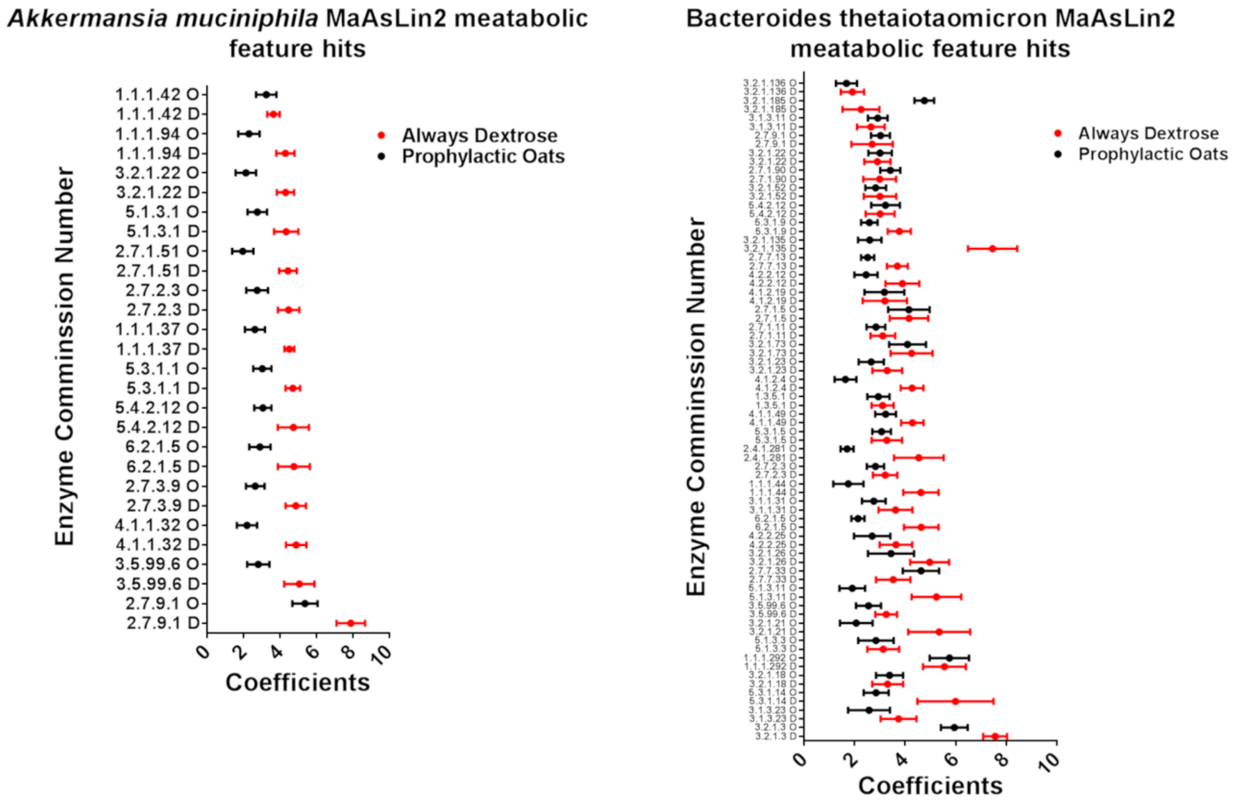

Supplementary Figure 4. Select metabolic features shared between diet conditions within *A. muciniphila* and *B. thetaiotaomicron* species. Select metabolic features relating to glycolysis/gluconeogenesis, the tricarboxylic acid cycle, the pentose phosphate pathway and simple and complex carbohydrate metabolism that were shared by the Always Dextrose and Prophylactic Oats diet groups within the A. *A. muciniphila* and B. *B. thetaiotaomicron* species. Each feature is represented as the model coefficient value (effect size) with model standard error from MaAsLin2 differential abundance analysis of *B. thetaiotaomicron* and *A. muciniphila* HUMAnN3 species level functional information (n = 4).
